# Negative association between triglyceride glucose index and BMI-adjusted skeletal muscle mass index in hypertensive adults

**DOI:** 10.1186/s12891-023-06700-7

**Published:** 2023-07-13

**Authors:** Qingqing Zhu, Ting Zhang, Iokfai Cheang, Xinyi Lu, Mengsha Shi, Xu Zhu, Shengen Liao, Rongrong Gao, Xinli Li, Wenming Yao

**Affiliations:** Department of Cardiology, Jiangsu Province Hospital and Nanjing Medical University First Affiliated Hospital, Guangzhou Road 300, Nanjing, 210029 China

**Keywords:** Triglyceride glucose index, Skeletal muscle mass index, NHANES, Hypertension

## Abstract

**Background:**

The triglyceride glucose (TyG) index, an indicator of insulin resistance, is often associated with adverse outcomes in various cardiovascular diseases, while hypertension is associated with an increased risk of cardiovascular diseases. As the loss of muscle mass in people with hypertension is poorly understood, the current study aimed to explore the relationship between TyG index and muscle mass in hypertensive population.

**Methods:**

We analyzed data from hypertensive adult participants in the National Health and Nutrition Examination Survey (NHANES) from 2011 to 2018. The TyG index and body mass index (BMI)-adjusted skeletal muscle mass index (SMI) were calculated and the relationship between the two was evaluated using multivariable linear regression and restricted cubic spline (RCS) regression models.

**Results:**

A total of 1633 participants in the dataset were included for the final analysis. In the multivariable regression analysis, the adjusted β of SMI with a 95% confidence interval (CI) for the highest TyG index quartile was − 5.27 (− 9.79 to − 0.75), compared with the lowest quartile. A negative linear relationship between TyG index and SMI was plotted by RCS regression (nonlinear *P* = 0.128). Stratified models of non-smoking women of different ages also demonstrated that SMI decreased as TyG index increased (all *P* for trend < 0.05).

**Conclusion:**

This linear and negative correlation between TyG index and SMI in hypertensive patients suggests that insulin resistance adversely affects muscle mass.

## Background

Hypertension currently affects approximately one-third of the global adult population. Despite considerable progress in pharmacotherapy, hypertension remains associated with an increased risk of cardiovascular disease (CVD) [1, 2], resulting in 9.4 million deaths (8.5% of the global total deaths) and 212 million health losses each year [3]. Studies on hypertension have predominantly focused on cardiovascular and cerebrovascular complications, often ignoring related muscle mass. The loss of muscle mass related to aging is known as primary sarcopenia. In contrast, secondary sarcopenia (or disease-related sarcopenia) is caused by diseases such as malignant cancer, chronic obstructive pulmonary disease, heart failure, and renal failure, and is primarily focused on loss of muscle mass rather than muscle function [4]. The low muscle mass and sarcopenia were highly prevalent in hypertensive when compared with non-hypertensive individuals [5, 6]. Currently, a multicenter, cross-sectional study showed a significantly decrease in the muscle mass in patients with hypertension compared with non-hypertensive patients across multiple different age ranges [7]. Meanwhile, a recent meta-analysis of large datasets showed that sarcopenia is significantly associated with hypertension [8]. Aging-related loss of skeletal muscle quantity and mass, with concomitant decreased vasodilatory capacity due to endothelial dysfunction, increases the risk of hypertension [9]. Reductions of skeletal muscle mass index (SMI) in participants were also associated with an increased risk of hypertension, and this was independent of other hypertension risk factors [10]. The etiology of the loss of skeletal muscle mass is not clear, nor is the regularity of changes affecting skeletal muscle mass in patients with hypertension. Therefore, exploration of the potential factors influencing skeletal muscle mass in a hypertensive population is warranted.

The triglyceride glucose index (TyG index) is a reliable surrogate marker of insulin resistance and an independent predictor of cardiovascular outcomes. A higher TyG index is associated with a higher risk of hypertension [11]. Additionally, previous cohort studies demonstrated that subjects with a higher TyG index may have a higher risk of arterial stiffness [12, 13]. Furthermore, a higher TyG index may be independently associated with a higher risk of atherosclerotic cardiovascular disease, coronary artery disease, and stroke [14], and it also serves as a valid predictor of adverse clinical outcomes in myocardial infarction patients [15].

The association of TyG index with the prevalence and prognosis of hypertension and multiple cardiovascular diseases warrants further studies to identify possible factors affecting sarcopenia in hypertension. Thus, we designed the present study to determine the potential association between TyG index and muscle mass based on cross-sectional data related to hypertension in the National Health and Nutrition Examination Survey (NHANES) database.

## Materials and methods

### Study population

Data used in this study were collected from NHANES 2011–2018, which is a public database conducted by the National Center for Health Statistics (NCHS) to evaluate the health status of the US general population. NHANES uses a complex, stratified and multistage probability sample to reflect the civilian non-institutionalized resident population information. The NHANES sample is drawn in four stages: (a) PSUs (counties, groups of tracts within counties, or combinations of adjacent counties), (b) segments within PSUs (census blocks or combinations of blocks), (c) dwelling units (DUs) (households) within segments, and (d) individuals within households. PSUs are sampled from all US counties. Screening is conducted at the DU level to identify sampled persons (SPs), based on oversampling criteria. In brief, NHANES sample design has consisted of multiyear, stratified, clustered four-stage samples, with public-use data release in 2-year cycles. Detailed study designs and procedures, including demographic, questionnaire, socioeconomic status, health status, health behavior, and examination results, are publicly available online (https://wwwn.cdc.gov/nchs/nhanes/Default.aspx).

**Fig. 1:**
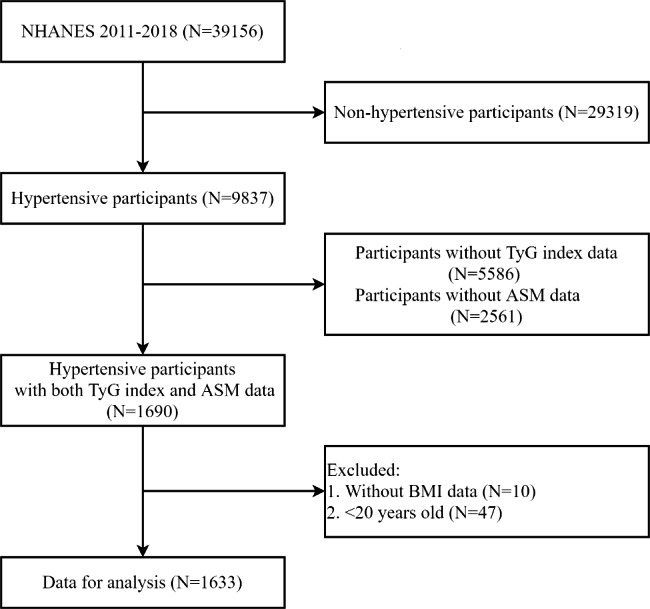
Flow chart of eligible participants in this study. NHANES, National Health and Nutrition Examination Survey; TyG index, triglyceride glucose index; ASM, appendicular skeletal mass; BMI, body mass index

In the present study, we integrated 8 years of data from continuous NHANES cohort (2011–2018). Among the 9837 total hypertensive participants in NHANES 2011–2018, those without TyG index (n = 5586) or appendicular skeletal mass (ASM) data (n = 2561) were excluded, resulting in 1690 available participants with hypertension plus TyG index and ASM data. We further excluded the participants without body mass index (BMI) data (n = 10) and under age 20 (n = 47), leaving a total of 1633 participants in the dataset for the final analysis. A flow chart of participant recruitment in the study is depicted in Fig. 1. The NHANES protocol was approved by the NCHS Ethics Review Board. All procedures met the criteria set out in the Declaration of Helsinki and all participants provided written informed consent.

### Definition of hypertension

Diagnosed hypertension was defined by a patient’s self-report of a history/physician’s diagnosis of hypertension or taking of anti-hypertensive agents in response to the questions “Have you/Has SP ever been told by a doctor or other health professional that you/s/he had hypertension, also called high blood pressure?” and “Have you/has s/he ever been told to take prescribed medicine?” Participants who answered “yes” to either of these two questions were defined as having hypertension. In addition, blood pressure measurements were also taken and used to confirm hypertension. Blood pressure was measured three times by a trained inspector using a mercury sphygmomanometer after the subjects rested quietly in a sitting position for 5 min. After calculating mean systolic (SBP) and diastolic blood pressure (DBP), diagnostic criteria were defined as SBP/DBP ≥ 140/90 mmHg in reference to the 2018 ESC/ESH or 2019 NICE guidelines [16, 17].

### Measurement of triglyceride glucose (TyG) index

The triglyceride glucose (TyG) index is the logarithmic product of fasting triglyceride and fasting glucose. The TyG index was calculated as ln [fasting triglyceride (mg/dL) × fasting glucose (mg/dL) / 2] [18]. In NHANES, serum triglyceride concentration was measured by an enzyme method using a Roche chemistry analyzer, and fasting plasma glucose was measured by hexokinase-mediated reaction.

### Measurement of BMI-adjusted skeletal muscle mass index (SMI)

SMI is defined as appendicular skeletal mass (ASM) of weight adjustment measured by dual energy X-ray absorptiometry (DXA). ASM is calculated as the sum of the muscle mass of the arms and legs, excluding the weight of bone and fat in the arms and legs. According to the criteria of the Foundation for the National Institutes of Health (FNIH) Sarcopenia Project, SMI in this study was defined as the ratio of ASM divided by BMI×100% [10, 19]. BMI is calculated by dividing body mass by stature squared (kg/m^2^).

### Covariates

This study summarizes potential covariates that may confound the association between TyG index and SMI in hypertensive populations. The variables, which were collected from an online questionnaire (http://www.cdc.gov/nchs/nhanes/nhanes_questionnaires.htm) included self-reported age, sex (male or female), race (Non-Hispanic White, Non-Hispanic Black, Mexican American, Other races), education level (less than high school, high school or equivalent, and college or above), energy intake, protein intake, carbohydrate intake, fat intake, poverty income ratio (PIR), and health behaviors including smoking (never, less than 100 cigarettes in their lifetime; former, those who had smoked more than 100 cigarettes but did not smoke at the time of survey; current, those who had smoked more than 100 cigarettes in their lifetime and smoked cigarettes at the time of the survey), alcohol consumption, and physical activity ((1) below, less than 600 MET min/week or 150 min/week of moderate-intensity exercise; (2) meet, 600 to 1200 MET min/week or 150 to 300 min/week of moderate-intensity exercise; or (3) exceed, at least 1200 MET min/week or 300 min/week of moderate-intensity exercise) [20]. Additionally, the plasma concentration of total cholesterol (TC) and estimated glomerular filtration rate (eGFR, calculated according to the chronic kidney disease-Epidemiology Collaboration equation) were also included [21].

### Statistical analysis

Given the complex sampling design of NHANES, the statistical analysis included sample weights, strata, and clustering to produce accurate nationally representative estimates [22]. Data in the present study were presented as weighted mean (standard error) for continuous variables and weighted percentages (standard error) for categorical variables. The study participants were modeled according to TyG quartiles into ordinal variables and divided into four groups. The multivariable linear regression model was used to determine the relationship between TyG index (both categorical and continuous variables) and SMI. In Model 1, the adjusted β was calculated for age, sex, and race. In Model 2, other potential confounding factors, including education level, PIR, smoking, alcohol use, physical activity, TC, and eGFR, were added. Based on Model 2, energy intake, protein intake, carbohydrate intake, and fat intake (Model 3) were comprehensively included to adjust for the effects of dietary intake on muscle mass.

Restricted cubic spline regression (RCS) was employed to plot the relationship between TyG index and SMI. RCS regression was adjusted for age, sex, race, education level, PIR, smoking, alcohol use, physical activity, TC, eGFR, dietary energy intake, protein intake, carbohydrate intake and fat intake (Model 3). In subgroup analysis, stratified multi-factor regression analysis was conducted according to age, sex, BMI, smoking, and physical activity to check whether the influence of SMI on TyG could be changed by these factors. An interaction term was added to examine the heterogeneity between subgroups. Sensitivity analyses of ASM/body weight as the outcome variable was also performed to validate the relationship between TyG index and SMI in hypertensive population.

All statistical analyses were weighted as recommended by NHANES and were conducted using STATA statistical software (version 14.0; StataCorp, College Station, TX, USA) and R software (version 3.6.0). A *P*-value (two-tailed) < 0.05 was defined as statistically significant.

## Results

### Baseline characteristics of participants with hypertension

Demographic characteristics of the study participants are shown in Table 1. A total of 1633 hypertensive adults were included in the study. The mean (SE) age of the participants was 46.1 (0.3) years and 54.0% were male. Age, sex, race, energy intake, protein intake, carbohydrate intake, fat intake, TC, body mass, stature, BMI, triglyceride, and fasting plasma glucose (FPG) were significantly different among groups with different levels of TyG index (all *P* < 0.05). No differences were observed in education level, smoking, alcohol consumption, physical activity, PIR, and eGFR among the four groups.

**Table 1:** Baseline characteristics of the study population (n = 1633), according to quartiles of TyG index

Characteristic	Overall (n = 1633)	Quartiles of TyG index				*P-*value
		Q1 (n = 408)	Q2 (n = 408)	Q3 (n = 409)	Q4 (n = 408)	
Age, years	46.1 (0.3)	44.6 (0.8)	45.6 (0.7)	46.6 (0.6)	47.6 (0.6)	0.007
Male, %	54.0 (1.4)	45.2 (3.6)	50.7 (2.7)	53.8 (3.6)	65.4 (2.4)	< 0.001
Race, %						< 0.001
Non-Hispanic White	62.5 (2.3)	54.2 (3.9)	61.8 (3.7)	62.0 (3.5)	71.2 (2.6)	
Non-Hispanic Black	16.1 (1.6)	29.2 (3.2)	16.9 (2.5)	12.9 (1.8)	6.2 (1.0)	
Mexican American	7.2 (1.0)	5.3 (1.2)	8.0 (1.5)	7.9 (1.5)	7.6 (1.4)	
Others	14.2 (1.1)	11.3 (1.8)	13.2 (1.6)	17.2 (2.1)	15.0 (1.8)	
Education level, %						0.294
Less than high school	15.9 (1.4)	13.2 (2.3)	14.9 (2.5)	20.2 (2.7)	15.5 (2.1)	
High school	24.5 (1.8)	27.4 (3.2)	25.0 (2.9)	25.6 (3.3)	20.4 (3.8)	
College or above	59.6 (2.1)	59.4 (3.4)	60.1 (3.4)	54.2 (4.1)	64.1 (3.8)	
Smoker, %						0.190
Never	50.3 (1.7)	58.1 (3.6)	47.6 (3.2)	47.8 (3.6)	48.2 (2.8)	
Former	23.4 (1.4)	17.3 (2.3)	26.3 (3.1)	25.4 (2.8)	24.1 (2.8)	
Current	26.3 (1.6)	24.6 (3.3)	26.1 (3.0)	26.8 (2.8)	27.7 (2.3)	
Alcohol user, %	84.0 (1.2)	87.2 (1.7)	82.7 (2.1)	83.0 (2.6)	83.2 (2.0)	0.367
Physical activity, %						0.205
Low	17.9 (1.1)	16.4 (2.4)	14.8 (2.4)	21.1 (2.9)	19.5 (2.5)	
Meet	12.2 (1.1)	10.3 (1.9)	11.9 (2.4)	10.3 (1.7)	16.1 (2.7)	
Exceed	69.9 (1.4)	73.3 (3.0)	73.3 (3.1)	68.6 (3.5)	64.4 (2.9)	
PIR	2.8 (0.1)	2.8 (0.1)	2.8 (0.1)	2.8 (0.1)	2.9 (0.1)	0.360
Energy intake, kcal/day	2317.7 (28.3)	2221.5 (62.0)	2270.1 (60.2)	2282.5 (78.1)	2486.4 (67.8)	0.012
Protein intake, g/day	88.0 (1.2)	84.6 (2.8)	84.8 (2.5)	87.0 (3.2)	95.2 (3.0)	0.002
Carbohydrate intake, g/day	269.3 (3.6)	256.6 (7.9)	261.7 (6.5)	270.3 (9.8)	287.4 (8.8)	0.013
Fat intake, g/day	89.9 (1.5)	83.7 (2.8)	88.5 (2.9)	89.0 (3.9)	97.7 (3.8)	0.008
Total cholesterol, mg/dL	196.8 (1.4)	179.9 (2.1)	191.6 (2.2)	200.7 (2.4)	214.0 (2.6)	< 0.001
eGFR, mL/min/1.73m^2^	99.4 (0.7)	100.6 (1.5)	98.9 (1.3)	100.6 (1.2)	97.7 (1.4)	0.312
Body mass, kg	93.0 (0.8)	84.5 (1.5)	92.8 (1.5)	94.6 (1.9)	99.5 (1.6)	< 0.001
Stature, cm	170 (0.3)	168.9 (0.7)	170.6 (0.6)	168.6 (0.8)	171.6 (0.6)	0.031
BMI, kg/m^2^	32.1 (0.3)	29.6 (0.5)	31.7 (0.4)	33.3 (0.6)	33.7 (0.5)	< 0.001
Triglyceride. Mg/dL	143.0 (4.2)	55.9 (1.0)	93.7 (1.2)	136.5 (2.0)	278.1 (10.5)	< 0.001
FPG, mg/dL	113.6 (1.3)	97.0 (0.7)	103.8 (0.9)	112.6 (2.1)	139.4 (4.0)	< 0.001

Data were presented as weighted mean (standard error) for continuous variables and weighted percentages (standard error) for categorical variables. All data originated from the National Health and Nutrition Examination Survey 2011–2018.

### A higher TyG index is associated with lower SMI

After fully adjusting for potential confounding factors, including age, sex, race, PIR, smoking, alcohol use, physical activity, energy intake, protein intake, carbohydrate intake, fat intake TC, and eGFR, a high TyG index (> 75th percentile) was associated with lower SMI (%) (β = −5.27, 95% CI: −9.79 to − 0.75), compared with the lowest quartile TyG index (*P* for trend = 0.016). Furthermore, the continuous analysis also revealed that a higher TyG index was associated with a lower SMI (β = −2.63, 95% confidence interval (CI): −4.77 to − 0.50) in the fully adjusted model (Model 3). The relationship between TyG index and SMI is shown in Table 2.

**Table 2:** Crude and adjusted β (95% CI) for associations between TyG index and SMI

TyG index	N^a^	Model 1	Model 2	Model 3
		β (95% CI)	β (95% CI)	β (95% CI)
Continuous	1633	-2.43 (-4.50, -0.37) *	-2.48 (-4.65, -0.32) *	-2.63 (-4.77, -0.50) *
Quartiles				
Q1	408	0 (Ref.)	0 (Ref.)	0 (Ref.)
Q2	408	-4.00 (-7.52, -0.49) *	-4.40 (-7.92, -0.89) *	-4.38 (-7.81, -0.96) *
Q3	409	-5.69 (-9.34, -2.04) **	-5.70 (-9.30, -2.09) **	-5.86 (-9.27, -2.44) **
Q4	408	-4.48 (-8.75, -0.21) *	-4.96 (-9.46, -0.46) *	-5.27 (-9.79, -0.75) *
*P* for trend		0.030	0.025	0.016

^a^Unweighted N.

TyG index: Q1 < 8.219, 8.219 ≤ Q2 < 8.681, 8.681 ≤ Q3 < 9.152, Q4 ≥ 9.152. * *P* < 0.05, ** *P* < 0.01. CI, confidence interval.

Model 1: adjusted for age, sex and race.

Model 2: adjusted for Model 1 plus education level, PIR, smoking, alcohol use, physical activity, TC and eGFR.

Model 3: adjusted for Model 2 plus dietary energy intake, protein intake, carbohydrate intake and fat intake.

**Fig. 2:**
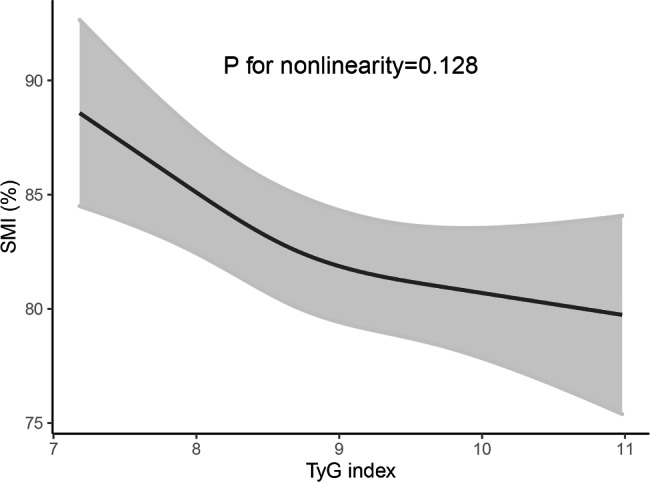
Restricted cubic spline (RCS) regression model between TyG index and SMI. The solid lines and shadow bands represent the corresponding SMI values and 95% confidence intervals

The continuous relationship between TyG index and SMI (ASM/BMI) based on the RCS model showed a linear and negative correlation between TyG index and SMI (nonlinear *P* = 0.128, Fig. 2).

### Subgroup analysis and sensitivity analysis

Subgroup analysis stratified by age, sex, smoking, BMI, and physical activity variables confirmed a negative association between TyG and SMI. The association between TyG index and SMI was similar across participant subgroups with no significant interactions between age (< 48 vs. ≥48 years), sex (male vs. female), BMI (≥ 30 vs. <30 kg/m2), and smoking status (Never vs. Former/Current) (*P* > 0.05 for all interactions). Women, non-smokers, and individuals whose physical activity met guideline recommendations had a higher risk of decreased muscle mass with increasing TyG index (*P* for trend = 0.002; *P* for trend = 0.018; *P* for trend = 0.012, respectively) (Table 3).

**Table 3:** Subgroups analysis for the association between TyG index and SMI

Subgroup	Q1		Q2	Q3	Q4	*P* for trend	*P* for interaction
	β		β (95%CI)	β (95%CI)	β (95%CI)		
**Age**							0.705
≥ 48 years	Ref.		-4.75 (-9.68, 0.18)	-5.96 (-9.77, -1.37) **	-6.20 (-11.78, -0.62) *	0.032	
< 48 years	Ref.		-4.10 (-8.88, 0.68)	-5.81 (-10.60, -1.02) *	-5.08 (-10.28, 0.13)	0.044	
**Sex**							0.784
Male	Ref.		-5.32 (-11.20, 0.55)	-6.24 (-12.86, 0.39)	-5.57 (-13.36, 2.22)	0.211	
Female	Ref.		-2.62 (-5.96, 0.72)	-5.32 (-9.23, -1.41) **	-5.17 (-8.85, -1.49) **	0.002	
**Smoke**							0.792
Never	Ref.		-1.21 (-4.89, 2.48)	-6.56 (-10.92, -2.20) **	-5.50 (-11.42, 0.43)	0.018	
Former/current	Ref.		-6.92 (-12.53, -1.32) *	-5.26 (-9.75, -0.77) *	-5.20 (-10.02, -0.38) *	0.168	
**BMI**							0.992
≥ 30 kg/m^2^	Ref.		-2.41 (-6.88, 2.05)	-2.22 (-6.28, 1.83)	-1.01 (-6.33, 4.31)	0.876	
< 30 kg/m^2^	Ref.		-2.84 (-7.25, 1.57)	-3.00 (-7.08, 1.08)	-1.72 (-6.69, 3.24)	0.392	
**Physical activity**							0.630
Low	Ref.		-2.08 (-7.63, 3.48)	-2.91 (-8.03, 2.22)	-2.76 (-10.12, 4.60)	0.447	
Meet	Ref.		-2.07 (-6.03, 1.89)	-2.77 (-6.59, 1.05)	-5.83 (-9.92, -1.74) **	0.012	
Exceed	Ref.		-5.03 (-9.41, -0.65) *	-6.86 (-11.51, -2.22) **	-5.49 (-11.48, 0.51)	0.058	

Analysis was adjusted for age, sex, race, education level, PIR, smoking, alcohol use, physical activity, TC, eGFR, dietary energy intake, protein intake, carbohydrate intake and fat intake (Model 3) when they were not the strata variables. * *P* < 0.05, ** *P* < 0.01. CI, confidence interval.

As a sensitivity analysis, when ASM/ body weight was defined as the outcome variable, a significant negative correlation was still observed between TyG index and SMI (ASM/ body weight) (Table 4). Compared with quartile 1 (the lowest quartile) of the TyG index, the adjusted β with 95% Cis of SMI (ASM/body weight) decreased [− 1.80 (− 2.94 to − 0.66), − 1.96 (− 3.02 to − 0.90), and − 2.07(− 3.46 to − 0.69) for quartiles 2, 3 and 4, respectively] across the increasing quartiles after fully adjusting for cofounding factors.

**Table 4:** Crude and adjusted β (95% CI) for associations between TyG index and SMI^a^.

TyG index	N^b^	Model 1	Model 2	Model 3
		β (95% CI)	β (95% CI)	β (95% CI)
Continuous	1633	-0.91 (-1.58, -0.24) **	-1.02 (-1.71, -0.32) **	-1.05 (-1.72, -0.37) **
Quartiles				
Q1	408	0 (Ref.)	0 (Ref.)	0 (Ref.)
Q2	408	-1.66 (-2.84, -0.48) **	-1.82 (-2.99, -0.66) **	-1.80 (-2.94, -0.66) **
Q3	409	-1.84 (-2.97, -0.71) **	-1.94 (-3.05, -0.83) **	-1.96 (-3.02, -0.90) ***
Q4	408	-1.73 (-3.07, -0.39) *	-2.02 (-3.43, -0.62) **	-2.07 (-3.46, -0.69) **
*P* for trend		0.014	0.006	0.004

^a^SMI was defined as ASM divided by body weight.

^b^Unweighted N.

TyG index: Q1 < 8.219, 8.219 ≤ Q2 < 8.681, 8.681 ≤ Q3 < 9.152, Q4 ≥ 9.152. * *P* < 0.05, ** *P* < 0.01, *** *P* < 0.001. CI, confidence interval.

Model 1: adjusted for age, sex and race.

Model 2: adjusted for Model 1 plus education level, PIR, smoking, alcohol use, physical activity, TC and eGFR.

Model 3: adjusted for Model 2 plus dietary energy intake, protein intake, carbohydrate intake and fat intake.

## Discussion

### Main findings and significance of this study

Using nationally representative NHANES data, this study demonstrated that SMI was negatively correlated with TyG index, and the relationship was linear.

Sarcopenia is associated with hypertension, particularly in the elderly patients [6, 8]. However, there is no clinical evidence to confirm the factors that may be related to decreased muscle mass in hypertensive patients. In recent Korean studies, TyG index was associated with low muscle mass [23, 24]; however, it has yet to be elucidated whether TyG index is related to SMI in other populations or in the presence of different diseases. The present study is the first to report the relationship between TyG index and muscle mass in US hypertensive patients. A population of 1633 individuals with hypertension in the United States was included in the current study, and TyG index was negatively correlated with SMI in hypertension, even after adjusting for age and other confounding factors. Given that TyG index is an indicator of insulin resistance, it suggests that patients with hypertension may be at greater risk of losing muscle mass as insulin resistance becomes more pronounced.

### Comparison of this study with previous studies

Recent clinical evidence proposed that a higher TyG index may be associated with a higher incidence of hypertension in the general adult population [11, 25, 26]. In addition, a prospective cohort study confirmed that TyG index was significantly associated with progression of arterial stiffness in hypertension [27]. Arterial disease is inseparable from sarcopenia, and the shared signaling pathways in peripheral arterial disease (PAD) and sarcopenia may involve oxidative stress, mitochondrial dysfunction, inflammation, impaired muscle synthesis, and degradation pathways [28]. Therefore, there may be an abnormal TyG index in hypertensive states, and this abnormal TyG index is potentially involved in the loss of muscle mass. The results from the current study are consistent with this theory.

### Potential mechanisms for the association between TyG index and muscle mass

The underlying biological mechanism of TyG index and muscle mass may be due to insulin resistance, inflammation, oxidative stress, and disorders of hormone regulation affecting muscle synthesis and catabolism. TyG index is an indicator of insulin resistance, consequently insulin resistance is not only independently associated with an increased risk of hypertension [29] but is also pathologically related to the loss of muscle mass. In the setting of insulin resistance, impaired insulin action in skeletal muscle leads to abnormal glucose uptake, dysregulated free fatty acid oxidation and inflamed adipose tissue spillover to ectopic sites, which ultimately impairs glucose homeostasis and protein synthesis, leading to accumulation of triglycerides in skeletal muscle and sarcopenia [30]. In vitro experiments revealed that induction of cellular insulin resistance increased the expression of muscle-specific E3 ligase MAFbx —the predominant mediator of skeletal muscle atrophy —in the C2C12 mouse myoblast cell line [31]. Furthermore, the RANK/RANKL system s instrumental in muscle metabolism, and inhibition of RANKL significantly improved muscle insulin sensitivity and reduced anti-myogenic and inflammatory gene expression in muscle, which potentially represents a novel therapeutic approach for sarcopenia [32]. Population-based studies have also identified that individuals with a higher muscle mass have enhanced insulin sensitivity compared with those with a lower muscle mass [33]. A study of 1098 patients with type 2 diabetes mellitus indicated that TyG index was inversely correlated with muscle mass [34]. The current study, with a larger sample size, yielded similar results and extended the association to the hypertensive population for the first time. Although the insulin resistance of patients with hypertension is not as severe as that of patients with diabetes, the conclusion remains the same and indicates the need to explore the underlying mechanisms to clarify the internal connections between muscle mass and hypertension.

Impaired muscle mass may also be associated with a complex interplay of factors such as oxidative stress, mitochondrial dysfunction, circulating pro-inflammatory cytokines, inflammatory macrophage infiltration of muscle tissue, and hormonal changes [35, 36]. Similarly, lipotoxicity, cytokines and macrophage inflammatory responses may all contribute to muscle insulin resistance [37]. Hormonal changes such as hyperleptinemia and leptin resistance directly interfere with skeletal muscle activity as well as disrupting the release of growth hormone, leading to insufficient anabolic effects on muscle and eventual muscle atrophy [38]. Furthermore, myokines are peptides secreted during skeletal muscle contraction that act through endocrine, paracrine, and autocrine pathways and affect skeletal muscle mass and insulin sensitivity [39–41]. However, the mechanisms by which myokines affect insulin resistance has not been fully elucidated, and further studies are also needed to explore the clinical role of myokines in hypertension and their clinical value in predicting impaired muscle mass.

### Strengths of this study

Based on the NIHANES project, which represents the largest continuous longitudinal population database in the United States, the present study employed the FNIH-recommended definition of sarcopenia using ASM adjusted by BMI and this provides epidemiological evidence in the relationship. In addition, we included eGFR alongside other common confounding factors such as demographic variables, metabolism, and nutrient intake owing to uremic sarcopenia being a common disorder in patients with chronic kidney disease and characterized by decreased muscle mass [42]. Ultimately, as the reduction of muscle mass is related to adverse cardiovascular outcomes [43], the negative correlation of TyG index with muscle mass provides a theoretical basis for the establishment of a sarcopenia prediction model in the future. In addition, these fundings may facilitate the development of clinical strategies to prevent impaired muscle mass.

### Limitations

The present study did have some limitations. Firstly, the cross-sectional design indicated that a causal relationship between TyG index and SMI could not be established. Secondly, the sample size was not large enough. The current study used the criteria for hypertension in 2018 ESC/ESH and 2019 NICE guidelines, rather than the diagnostic criteria in the 2017 ACC/AHA guidelines, which prevented overdiagnosis [44] but also restricted the sample size. Thirdly, there was a possibility of selection bias because we could only select participants with DXA examination, fasting triglyceride and glucose simultaneously in this study. Finally, although SMI is a convenient and effective tool for assessing skeletal muscle, it should be noted that in remote and economically underdeveloped regions, the obtaining of SMI will increase the cost in data collection. Since NHANES was previously collected data and was not designed to meet the objectives of the study, further validation in other subpopulations will be useful to determine the representation of our findings.

## Conclusion

This study revealed a linear and negative relationship between TyG index and BMI-adjusted skeletal muscle mass index in individuals with hypertension. Further validation of this result in prospective cohort studies is required.

## Data Availability

The data for this study can be found in the National Health and Nutrition Examination Survey (https://wwwn.cdc.gov/nchs/nhanes/Default.aspx).

## Abbreviations

ASM: Appendicular skeletal mass
BMI: Body mass index
CI: Confidence interval
CVD: Cardiovascular disease
DBP: Diastolic blood pressure
eGFR: Estimated glomerular filtration rate
FPG: Fasting plasma glucose
NHANES: National Health and Nutrition Examination Survey
PIR: Poverty income ratio
RCS: Restricted cubic spline
SBP: Systolic blood pressure
SMI: Skeletal muscle mass index
TC: Total cholesterol
TyG index: Triglyceride glucose index
